# Hyperammonaemia induces mitochondrial dysfunction and neuronal cell death

**DOI:** 10.1016/j.jhepr.2022.100510

**Published:** 2022-05-23

**Authors:** Plamena R. Angelova, Annarein J.C. Kerbert, Abeba Habtesion, Andrew Hall, Andrey Y. Abramov, Rajiv Jalan

**Affiliations:** 1 Queen Square Institute of Neurology, University College London, London, UK; 2 Institute for Liver and Digestive Health, University College London, Royal Free Campus, London, UK; 3 Sheila Sherlock Liver Centre, Royal Free London NHS Foundation Trust, London, UK; 4 European Foundation for the Study of Chronic Liver Failure, Barcelona, Spain

**Keywords:** Liver cirrhosis, Hepatic encephalopathy, Ammonia, Mitochondrial function, Cell death, Δψm, mitochondrial membrane potential, BDL, bile duct ligation, CA1, cornu ammonis 1, CA3, cornu ammonis 3, CNS, central nervous system, DG, dentate gyrus, DIV, days *in vitro*, ETC, electron transport chain, FCCP, carbonyl cyanide 4-(trifluoromethoxy) phenylhydrazone, GFAP, glial fibrillary acidic protein, HE, hepatic encephalopathy, HEt, dihydroethidium, LP, lipid peroxidation, mPT, mitochondrial permeability transition, NaCN, sodium cyanide, OP, ornithine phenylacetate, PI, propidium iodide, ROI, region of interest, ROS, reactive oxygen species, TCA, tricarboxylic acid, TMRM, tetramethyl rhodamine, methyl ester

## Abstract

**Background & Aims:**

In cirrhosis, astrocytic swelling is believed to be the principal mechanism of ammonia neurotoxicity leading to hepatic encephalopathy (HE). The role of neuronal dysfunction in HE is not clear. We aimed to explore the impact of hyperammonaemia on mitochondrial function in primary co-cultures of neurons and astrocytes and in acute brain slices of cirrhotic rats using live cell imaging.

**Methods:**

To primary cocultures of astrocytes and neurons, low concentrations (1 and 5 μM) of NH_4_Cl were applied. In rats with bile duct ligation (BDL)-induced cirrhosis, a model known to induce hyperammonaemia and minimal HE, acute brain slices were studied. One group of BDL rats was treated twice daily with the ammonia scavenger ornithine phenylacetate (OP; 0.3 g/kg). Fluorescence measurements of changes in mitochondrial membrane potential (Δψm), cytosolic and mitochondrial reactive oxygen species (ROS) production, lipid peroxidation (LP) rates, and cell viability were performed using confocal microscopy.

**Results:**

Neuronal cultures treated with NH_4_Cl exhibited mitochondrial dysfunction, ROS overproduction, and reduced cell viability (27.8 ± 2.3% and 41.5 ± 3.7%, respectively) compared with untreated cultures (15.7 ± 1.0%, both *p* <0.0001). BDL led to increased cerebral LP (*p* = 0.0003) and cytosolic ROS generation (*p* <0.0001), which was restored by OP (both *p* <0.0001). Mitochondrial function was severely compromised in BDL, resulting in hyperpolarisation of Δψm with consequent overconsumption of adenosine triphosphate and augmentation of mitochondrial ROS production. Administration of OP restored Δψm. In BDL animals, neuronal loss was observed in hippocampal areas, which was partially prevented by OP.

**Conclusions:**

Our results elucidate that low-grade hyperammonaemia in cirrhosis can severely impact on brain mitochondrial function. Profound neuronal injury was observed in hyperammonaemic conditions, which was partially reversible by OP. This points towards a novel mechanism of HE development.

**Lay summary:**

The impact of hyperammonaemia, a common finding in patients with liver cirrhosis, on brain mitochondrial function was investigated in this study. The results show that ammonia in concentrations commonly seen in patients induces severe mitochondrial dysfunction, overproduction of damaging oxygen molecules, and profound injury and death of neurons in rat brain cells. These findings point towards a novel mechanism of ammonia-induced brain injury in liver failure and potential novel therapeutic targets.

## Highlights

• Low concentrations of ammonia induce mitochondrial dysfunction, overproduction of ROS, and cell death in primary neurons.
• Hyperammonaemia in cirrhotic rats leads to ROS and LP overproduction, which was prevented by the ammonia scavenger OP.
• In neurons from cirrhotic rats, hyperpolarisation of Δψm was observed, which was restored by OP treatment.
• In a rat model of cirrhosis, profound neuronal loss was observed in the hippocampus.

## Graphical abstract

**Figure:**
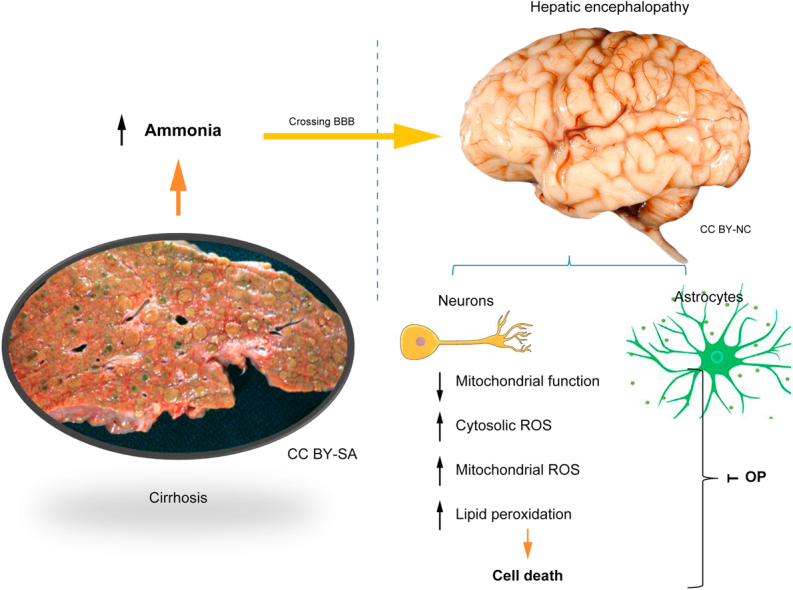

## Introduction

Hepatic encephalopathy (HE) is a severe complication of cirrhosis and manifests with a wide range of cognitive, psychiatric, and motor system abnormalities.^1^ In patients with liver failure, hepatic detoxification of ammonia via the urea cycle is impaired, which leads to increased ammonia levels in the circulation.^2^ Ammonia in its gaseous form can freely pass the blood–brain barrier, where it is known to induce a variety of derangements in the central nervous system (CNS), including astrocyte swelling, brain oedema, neuroinflammation, increased glutamatergic neurotransmission, and oxidative.^3^^,^^4^

As the CNS is highly dependent on mitochondrial energy supply in the form of ATP, alteration of the energy balance of neurons that leads to energy deficit and oxidative stress ultimately initiates CNS injury and cell death.^5^^,^^6^ In the last decade, studies have suggested a possible role for ammonia-induced mitochondrial dysfunction in the development of HE.[7], [8], [9] The exact mechanism has not yet been fully elucidated, but it includes glutamine-derived ammonia accumulation within the mitochondrial matrix of astrocytes, ammonia-induced inhibition of tricarboxylic acid (TCA) cycle enzymes, induction of mitochondrial permeability transition (mPT), impairment of electron transport chain complexes, and induction of oxidative stress.^7^^,^^10^ However, the data from these studies are difficult to put into clinical context as almost all these studies have used *in vitro* models with non-physiologically high concentrations of ammonia and focussed almost entirely on the astrocytes.^11^

Brain energy metabolism is a well-balanced process that encompasses not only the processes of ATP production inside the cells but also neuron–glia interactions.^5^ Considering this, even if ammonia has a direct effect only on astrocytes, energy metabolism in neurons is unlikely to remain unaffected. Traditionally, HE has been thought to be reversible, but emerging data clearly indicate that complete recovery of cognitive function is not consistently observed in patients with an episode of HE,^12^^,^^13^ suggesting that neuronal dysfunction and cell death may be important in the pathogenesis of HE. In this study, we aimed to investigate the impact of low-grade hyperammonaemia on mitochondrial function in neurons and astrocytes both *in vitro* (primary co-cultures of neurons and astrocytes) and *ex vivo* (acute brain slices) using live cell imaging techniques. We have found that even disease-relevant concentrations of ammonia significantly alter the mitochondrial metabolism of primary neurons, activates overproduction of reactive oxygen species (ROS) and oxidative stress that leads to neuronal cell death. Importantly, in acute brain slices of a rodent model of HE, we also observed mitochondrial dysfunction, ROS overproduction, and loss of neurons in different hippocampal areas. Importantly, ammonia-lowering treatment with ornithine phenylacetate (OP) led to the prevention of hyperpolarisation of the mitochondrial membrane potential (Δψm) and reduced ROS production in neurons and thereby also had a neuroprotective effect.

## Materials and methods

All experimental procedures were performed in compliance with the United Kingdom Animal (Scientific Procedures) Act of 1986 (updated 2012) and with the European directive 2010/63/EU, approved by the University College London (UCL) Animal Welfare and Ethical Review Body under full project and establishment licenses (No. 14378). Rats were group housed in individually ventilated cages and kept on a 12-h light–dark cycle with *ad libitum* access to water and food.

### Cell culture

Primary co-cultures of neurons and astrocytes were isolated from brains of Sprague Dawley pups from the UCL breeding colony (P3-4) following the method described by Angelova *et al.*^14^ with few modifications. In brief, the brain was extracted, homogenised, and dissociated with 0.25% trypsin-EDTA (Sigma-Aldrich). The mixed neuron and astrocyte suspension was plated on precoated glass coverslips with poly-d-lysine (1 mg/ml). Cells were maintained in Neurobasal with 2% of B27 (Invitrogen) and 2 mM l-glutamine, 1% of penicillin/streptomycin (Sigma-Aldrich). Cell cultures were maintained in a humidified incubator at 37°C and 5% CO_2_.

As the *in vitro* environment of neuronal cultures lacks important buffering and metabolic pathways, we decided to start with very low concentrations of NH_4_Cl in the *in vitro* studies (1 and 5 μM NH_4_Cl) to prevent inducing cell death straight away before or during live cell imaging. Surprisingly, we already found an effect on mitochondrial function with these low concentrations. Therefore, we decided to proceed with these concentrations also in the *ex vivo* brain slices studies, which showed consistent results.

To assess the age dependency of NH_4_Cl effects, 2 "age" groups were used: 7 days *in vitro* (DIV, *i.e.* immature cultures) and 16DIV (*i.e.* mature cultures). We have chosen to study these 2 time points, as we have shown in previous studies that there is a profound difference in mitochondrial bioenergetics between immature and mature neurons.^15^ Neurons were easily distinguishable from glia: they appeared phase bright, had smooth rounded somata and distinct processes, and laid just above the focal plane of the glial layer.

### Rodent model of hepatic encephalopathy

In male Sprague Dawley rats (age 8–10 weeks, weight ∼300–450 g), liver cirrhosis was induced by bile duct ligation (BDL) surgery, as described by Harry *et al.*^16^ Rats were studied 4 weeks after BDL (N = 5) or sham (N = 5) operation. Of the BDL-operated rats, 2 were treated with the ammonia-scavenging drug OP (0.3 g/kg i.p.) twice daily during the last 5 days of the model.

### Acute brain slices

Rats were sacrificed by neck dislocation under general anaesthesia (2% isoflurane in oxygen) after a cardiac perfusion with cold Ringer solution. Following decapitation, brains were rapidly removed, and transverse slices (100 μm thick) were prepared using a Leica VT1200S vibratome. Slicing was performed in ice-cold solution that contained (in mM) 120 NaCl, 10 glucose, 2.5 KCl, 1.3 MgSO_4_, 1 NaH_2_PO_4_, 1.3 MgCl_2_, 2 CaCl_2_, and 10 HEPES, with pH adjusted to 7.4. Once cut, slices were incubated at room temperature for 1 h for recovery in the presence of fluorescent indicators.

### Live cell imaging

Fluorescence measurements of changes in Δψm, mitochondrial ROS production, rate of lipid peroxidation (LP) and assessment of cell death were performed using a Zeiss 710 visible confocal laser scanning microscopy (VIS CLSM) equipped with a META detection system and a 40× oil immersion objective.

#### Mitochondrial membrane potential

Tetramethyl rhodamine, methyl ester (TMRM) is a cationic, cell-permeant fluorescent dye that was used to assess Δψm. The cells and slices were loaded with 25 nM TMRM for 40 min. Z-stacks were acquired using an excitation wavelength of 561 nm with a long pass filter and a 40× oil immersion objective.

#### Mitochondrial ROS production

For assessing mitochondrial ROS production, cells and slices were loaded with 1 μM MitoTracker Red CM-H2Xros (Thermo Scientific) and incubated for 20 min, after which the increase in fluorescence over time was imaged using 561-nm excitation and a long-pass filter. Confocal images were obtained using a 40× oil immersion objective.

#### LP

For assessing the LP, cells and slices were loaded with 5 μM C11-BODIPY (581/591) for 30 min and washed. The dye was excited at 488 and 561 nm and detected using a 40× oil immersion objective. The ratio of 581/591 nm was analysed, and the rates were then calculated in AU/min.

#### Cell death

For assessment of cell death *in vitro*, cells were treated for 24 h with 1 and 5 μM NH_4_Cl. Before imaging, cells were incubated with propidium iodide (PI; 10 μM) and 300 nM Hoechst for 15 min, washed 3 times with PBS 1× and analysed using a cooled charge-coupled device (CCD) camera. Hoechst stains the total number of nuclei, whereas PI stains only cells with a disrupted plasma membrane. Dead cells (PI positive) were counted as a fraction of the total (Hoechst positive). In each experiment, 5 random fields were examined. The mean is representative for 3 independent experiments for each condition.

#### Cytosolic ROS production

Superoxide production was measured by using dihydroethidium (HEt; 5 μM, Invitrogen) in HBSS at room temperature. Fluorescent images were acquired using an inverted epifluorescence microscope, equipped with a 20× fluorite objective at a frame interval of 10 s. The ratio of oxidised and reduced forms of HEt was measured at 530-nm excitation and emission above 560 nm to allow quantification of the oxidised form (ethidium), whereas 380-nm excitation and emission from 405 to 470 was used to record the reduced form of the dye. Data were analysed using software from Andor IQ3 (Andor Technology, Belfast, UK).

#### NADH redox index and NADH pools

The autofluorescence of NADH and NADPH (which can be referred to as NAD(P)H) was imaged on a cooled CCD camera (Hamamatsu, Orca ER). The blue autofluorescence, emitted by the pyridine nucleotides NADH and NADPH in their reduced form, was excited using a 360-nm filter and emission was collected using a 455-nm filter. Confocal images were acquired using a Zeiss 510 UV laser scanning microscope system and a 40× objective. The application of 1 μM of the mitochondrial uncoupler carbonyl cyanide 4-(trifluoromethoxy) phenylhydrazone (FCCP) maximised the rate of respiration and oxidised the mitochondrial NADH pool in cells, resulting in a decrease of detected fluorescence (minimum = 0% for NADH). The subsequent application of 1 mM of the complex IV inhibitor sodium cyanide (NaCN) suppressed respiration, preventing NADH oxidation and allowing the NADH pool to be regenerated (maximum = 100% for NADH).^17^ Quantitative analysis of the obtained images was performed cell by cell using the Andor IQ3 software (Andor Technology). The average was taken from n >3 independent experiments for each condition.

### Immunofluorescence

Glial fibrillary acidic protein (GFAP) and beta III tubulin immunofluorescence staining was performed in the paraffin-embedded brain tissue of the BDL rat model to stain, respectively, astrocytes and neurons. Tissue sections were dewaxed with xylene (15 min ×3) and hydrated through ethanol (2 min ×3 to tap water). Sections were microwaved at 640 W in 1 L citrate buffer (10 mM sodium citrate, 0.05% Tween 20, pH 6.0) for 20 min, after which a protein block was applied (Abcam ab64226) for 10 min. Sections were then incubated in a primary antibody with mouse Anti-beta III Tubulin (Abcam ab78078) and rabbit anti-GFAP antibody (Abcam ab7260), 1:200 and 1:1,000, respectively, diluted in antibody diluent (Agilent S080983-2). After incubation in the primary antibody, sections were washed in TBS pH 7.6 (5 min) before the application of secondary antibodies: goat anti-mouse IgG-Alexa Fluor 594 (ab150120) and goat anti-rabbit IgG-Alexa Fluor 647 (Abcam ab150079) both at 1:100 dilution diluted in TBS pH 7.6 for 30 min in the dark at room temperature. Sections were mounted in an aqueous mounting medium with DAPI (Abcam ab1044139). A multispectral image of each slide was taken using an Akoya Mantra 2 imaging system. A 14-bit-depth image was taken at 10-nm intervals between 400 and 720 nm for each of 7 different filters, DAPI, CYP, FITC, CY3, TEXRED, CY5, and CY7, and spectrally unmixed.

Immunoreactivity image analysis was performed using ImageJ software (1.51j8). Cell layer densities of the dentate gyrus (DG), cornu ammonis 1 (CA1), and cornu ammonis 3 (CA3) hippocampal regions of individual animals were calculated as the average densities of each region obtained from 15 regions of interest (ROIs) per area per slice. Results are expressed as integrated density taken from the mean fluorescence intensity (in pixels) per μm^2^.

### Statistical analysis

Data were analysed using Origin Pro 2019 (MicroCal, Oregon, USA) and are expressed as mean ± SEM, number of animals (N), and number of slices (n), unless otherwise stated. Two-tailed unpaired Student’s *t* test or 1-way ANOVA followed by the Bonferroni *post hoc* test was used to estimate the statistical significance between experimental groups. Significance was accepted at a 95% CI (*p* <0.05; ∗*p* <0.05, ^†^*p* <0.001, ^‡^*p* <0.0001).

## Results

### Cell culture

#### Micromolar concentrations of ammonium chloride induced mitochondrial dysfunction in primary neurons

**Fig. 1:**
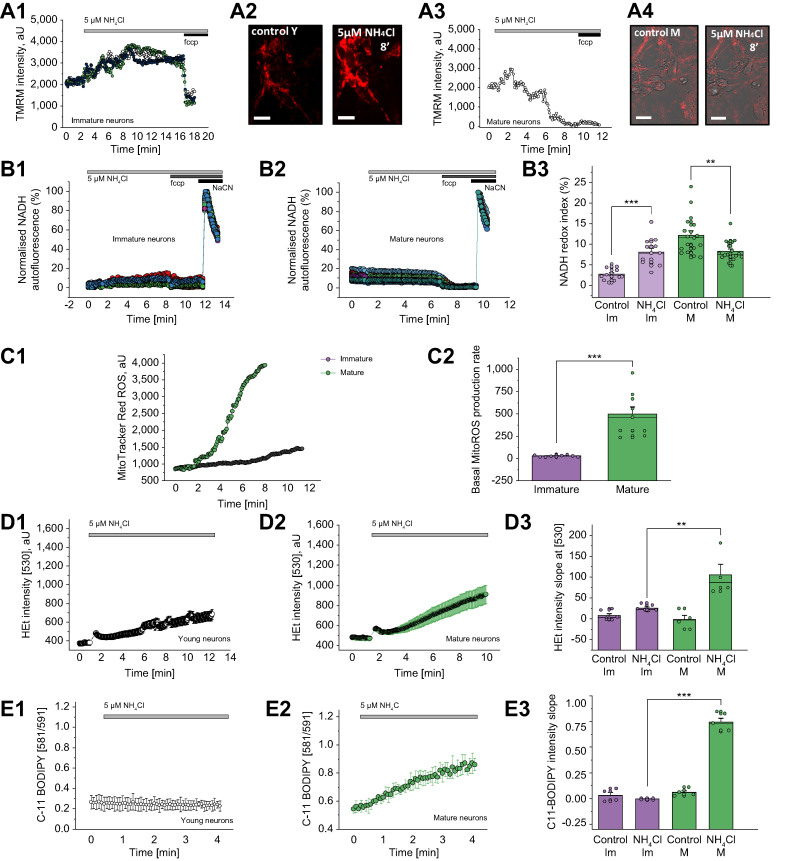
Age-dependent NH_4_Cl effect on mitochondrial function and oxidative status of primary cultures. (A) Kinetic changes in ΔΨm from immature (A1) and mature (A3) rat primary neurons. Representative TMRM images before and 8 min after the application of 5 μM NH_4_Cl in immature (A2) and mature (A4) primary neurons. (B) Changes in mitochondrial respiration of immature (A1) and mature (A1) rat neurons upon the application of 5 μM NH_4_Cl. (A3) Quantification bar chart of the results in A1 and A2. (C) Age-dependent mitochondrial ROS production. Representative traces (C1) and quantification bar chart of basal rate of mitochondrial ROS generation rate (C2). (D) Cytosolic ROS production in immature (D1) and mature (D2) primary rat neurons. Quantification of the results (D3) from D1 and D2. (E) Lipid peroxidation in immature (E1) and in mature (E2) neurons from rat primary culture. Quantification of the results (E3) from E1 and E2. Scale bar = 50 μm. Data are represented as mean ± SEM. ∗*p* <0.05, ∗∗*p* <0.001, ∗∗∗*p* <0.0001, two-tailed unpaired Student’s *t* test. ΔΨm, mitochondrial membrane potential; FCCP, carbonyl cyanide 4-(trifluoromethoxy) phenylhydrazone; HEt, dihydroethidium; Im, immature; M, mature; NaCN, sodium cyanide; ROS, reactive oxygen species; TMRM, tetramethyl rhodamine, methyl ester.

In a set of experiments in primary co-cultures, we were able to control for NH_4_Cl delivered directly to neurons and astrocytes. The effect of 5 μM NH_4_Cl was found to be age-dependent. First, we assessed Δψm, which is involved in most mitochondrial processes and can be taken as an indicator of mitochondrial health. In immature neurons (7DIV), the application of NH_4_Cl induced hyperpolarisation of the mitochondrial membrane (Fig. 1A1). In mature neurons (16DIV), however, the application of NH_4_Cl induced a profound mitochondrial depolarisation (Fig. 1A3).

NADH is a substrate and donor of electrons for complex I of mitochondria, and changes in NADH autofluorescence can help estimate the activity of NADH-dependent respiration in living neurons and astrocytes. The application of 5 μM NH_4_Cl to immature neurons induced a moderate increase in NADH autofluorescence (Fig. 1B1 and B3, from 2.67 ± 1.45% to 8.03 ± 3.36%, n = 12, *p* <0.0001) that may be explained by the activation of NADH production in the TCA cycle or by a mild inhibition of mitochondrial respiration. In mature primary neurons, the addition of 5 μM NH_4_Cl induced a slow decrease in NADH autofluorescence. This, in combination with the data obtained for the Δψm, strongly suggests mitochondrial uncoupling (Fig. 1B2 and B3, from 12.17 ± 4.63% to 8.26 ± 2.47%, n = 18, *p* = 0.0033).

#### Micromolar concentrations of ammonium chloride induced mitochondrial ROS production and oxidative stress in primary neurons

The effects of 5 μM exogenously applied NH_4_Cl on mitochondrial ROS production also appeared to be dependent on age: immature neurons produced less mitochondrial and cytosolic ROS than did mature neurons (mitochondrial: 23.93 ± 5.88 *vs*. 70.30 ± 48.91, n = 9, *p* = 0.0122; Fig. 1C1 and C2; cytosolic: 25.92 ± 7.34 *vs.* 104.97 ± 52.61, n = 9, *p* = 0.0057; Fig. 1D1–D3). However, ROS production can play a physiological role, and in cells with an efficient antioxidant system, it does not induce oxidative damage. The LP rate, 1 of the hallmarks of oxidative stress, indicates possible damage to cell lipid composition that is involved in neuronal pathology. Importantly, 5 μM NH_4_Cl induced more activation of LP in mature neurons than in immature ones (0.0051 ± 0.009 *vs*. 0.7428 ± 0.091, *p* <0.0001; Fig. 1E1–E3). Thus, micromolar concentrations of ammonia induced overproduction of ROS in mitochondria and the cytosol, which led to LP and oxidative stress.

### Acute brain slices

We have previously shown, in large numbers of sham (n = 22), BDL (n = 26), and BDL + OP-treated (n = 14) Sprague Dawley rats, that BDL rats have significantly higher plasma ammonia levels than sham (141 ± 4 μmol/L *vs*. 56 ± 3 μmol/L, *p* <0.001), which is normalised by OP treatment (60 ± 2 μmol/L; Table S1).^18^

#### Hyperammonaemia leads to hyperpolarisation of brain mitochondria

**Fig. 2:**
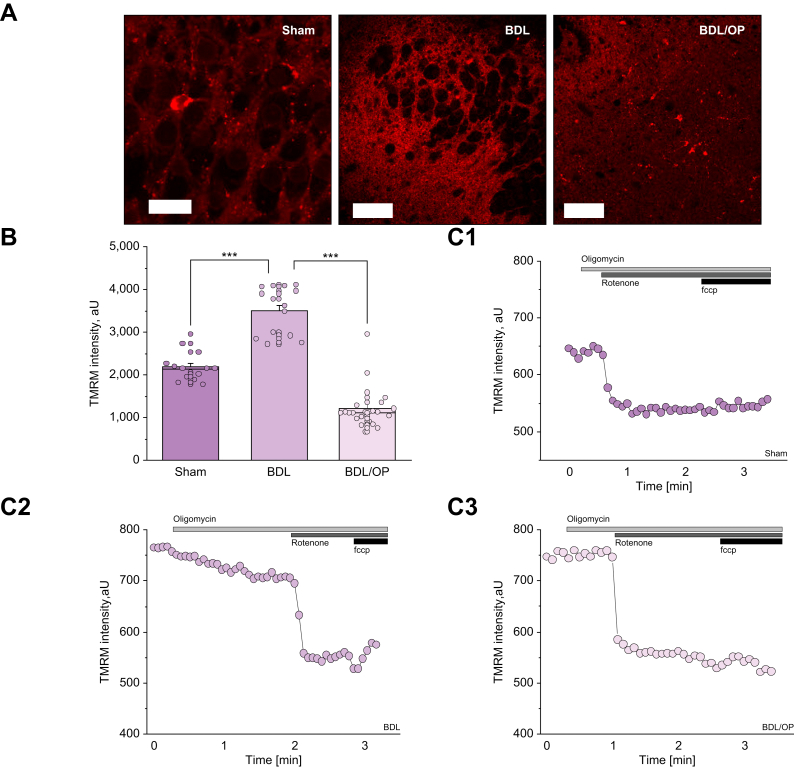
BDL is associated with hyperpolarisation of ΔΨm, which is restored by OP treatment. (A) Representative images of TMRM fluorescence in acute brain slices from sham-operated, BDL, and BDL/OP rats. (B) Quantification bar-chart of ΔΨm in acute slices from sham-operated, BDL, and BDL/OP rats. (C) representative traces of dynamic changes of TMRM intensity from sham-operated (C1), BDL (C2), and BDL/OP (C3) rat brain slices upon the application of oligomycin (2 μg/ml), rotenone (1 μM) and FCCP (1 μM). Scale bar = 50 μm. Data are represented as mean ± SEM. ∗*p* <0.05, ∗∗*p* <0.001, ∗∗∗*p* <0.0001, two-tailed unpaired Student’s *t* test. ΔΨm, mitochondrial membrane potential; BDL, bile duct ligation; FCCP, carbonyl cyanide 4-(trifluoromethoxy) phenylhydrazone; OP, ornithine phenylacetate; TMRM, tetramethyl rhodamine, methyl ester.

Using TMRM as a fluorescent indicator for Δψm in acute brain slices, we observed that hyperammonaemia led to hyperpolarisation of mitochondria (sham: 2,181.64 ± 90.76, n = 14, *vs*. BDL: 3,482.00 ± 554.44, n = 15, *p* <0.0001; Fig. 2B and C1 and C2). Scavenging of the ammonia by administration of OP to BDL animals effectively reduced Δψm in acute brain slices. Interestingly, OP-treated BDL animals had even lower Δψm values than sham animals (Fig. 2B and C3, from 3,482.00 ± 143.16, n = 15 for BDL to 1,205.39 ± 99.63, n = 23 for BDL + OP, *p* <0.0001).

Hyperpolarisation or depolarisation of mitochondria could be induced by various triggers. To understand the mechanism of the effect of BDL on mitochondrial function, we applied several mitochondrial toxins (*i.e.* oligomycin, rotenone, and FCCP) in the TMRM measurements. As expected, the application of 2 μg/ml oligomycin, an inhibitor of ATP synthase/ATPase, had no effect on Δψm in sham animals (Fig. 2C1, n = 14). The application of the complex I inhibitor rotenone (5 μM) induced complete mitochondrial depolarisation, and the uncoupler FCCP (1 μM) did not induce a further decrease in TMRM fluorescence. This strongly suggests that Δψm of the cells in these brain slices are exclusively maintained by the electron transport chain (ETC) of mitochondria. In acute brain slices from BDL rats, the application of oligomycin induced a 25% decrease in Δψm (Fig. 2C2, n = 15) suggesting that owing to dysfunction in ETC, Δψm is maintained by the consumption of ATP in the ATPase instead of its production. However, rotenone still induced a profound decrease in TMRM fluorescence, indicating that part of the Δψm is still maintained by the ETC. Such combination of mitochondrial respiration and ATPase activity leads to pathological mitochondrial hyperpolarisation.^15^^,^^19^^,^^20^ Importantly, the administration of OP led to recovery of the response to oligomycin, but this was not sufficient to restore the Δψm to control levels (Fig. 2C3, n = 23).

#### Hyperammonaemia led to overproduction of ROS in neuronal mitochondria

**Fig. 3:**
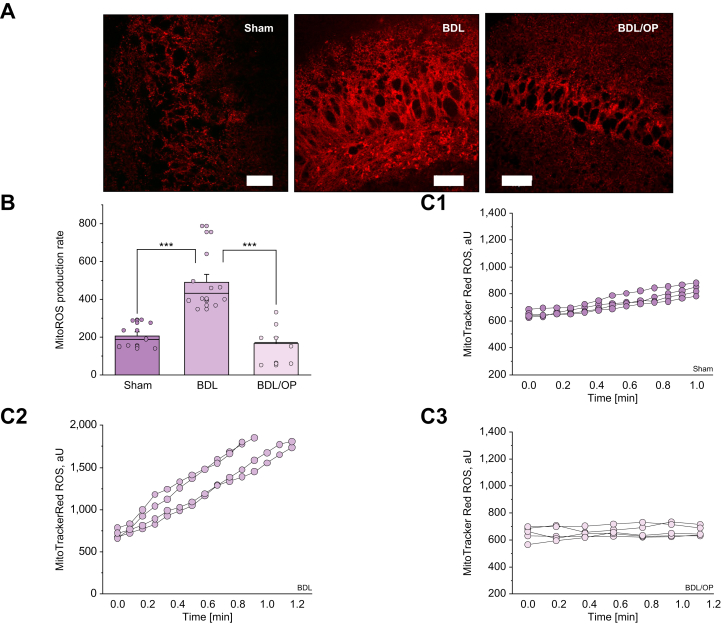
Mitochondrial ROS production is increased in BDL and mitigated by OP. (A) Representative images of MitoTracker Red CM-H2Xros fluorescence in acute slices from sham-operated, BDL, and BDL/OP rats. (B) Quantification bar chart of mitochondrial ROS production rate in acute slices from sham-operated, BDL, and BDL/OP rats. (C) Representative traces of several ROIs of MitoTracker Red CM-H2Xros intensity from sham-operated (C1), BDL (C2), and BDL/OP (C3) brain slices. Scale bar = 50 μm. Data are represented as mean ± SEM. ∗*p* <0.05, ∗∗*p* <0.001, ∗∗∗p <0.0001, two-tailed unpaired Student’s *t* test. BDL, bile duct ligation; OP, ornithine phenylacetate; ROS, reactive oxygen species.

Hyperpolarisation of mitochondria in combination with altered mechanism of Δψm maintenance can lead to the activation of ROS generation in the ETC.^6^^,^^19^^,^^20^ To test if increased Δψm in BDL rats can trigger ROS production, we measured the rate of mitochondrial ROS production in acute brain slices using MitoTracker ROS. The rate of mitochondrial ROS production in brain slices from BDL rats was 2.6 times higher than in those of control rats (205.3 ± 21.8, n = 9, for control *vs*. 487.1 ± 44.64 for BDL, n = 12, *p* <0.0001; Fig. 3A, B, and C1 and C2). Importantly, the administration of OP reduced the production of ROS in mitochondria from BDL rats to levels seen in the controls (from 487.1 ± 44.64, n = 12, for BDL to 162.6 ± 36.6, n = 8, for BDL + OP, *p* <0.0001).

#### Hyperammonaemia induces intracellular ROS overproduction

**Fig. 4:**
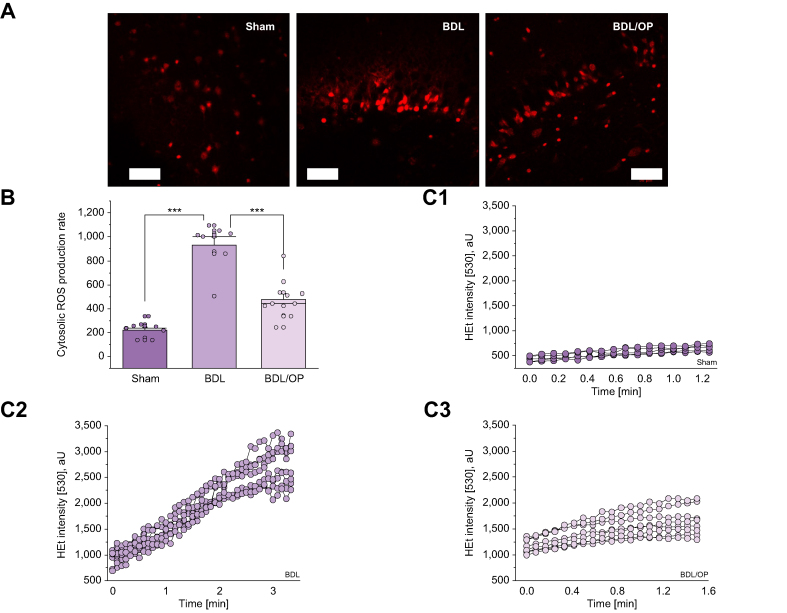
Cytosolic ROS production is elevated in BDL and alleviated by OP. (A) Representative images of HEt fluorescence in acute slices from sham-operated, BDL, and BDL/OP rats. (B) Quantification bar chart of superoxide production rate in acute slices from sham-operated, BDL, and BDL/OP rats. (C) Representative traces of several ROIs of HEt intensity at 530 nm from sham-operated (C1), BDL-subjected (C2), and BDL/OP (C3) slices. Scale bar = 50 μm. Data are represented as mean ± SEM. ∗*p* <0.05, ∗∗*p* <0.001, ∗∗∗*p* <0.0001, two-tailed unpaired Student’s *t* test. BDL, bile duct ligation; HEt, dihydroethidium; OP, ornithine phenylacetate; ROI, region of interest; ROS, reactive oxygen species.

Mitochondria are just one of the multiple sources of ROS production in brain cells. Additionally, mitochondria produce superoxide anion and hydrogen peroxide into the matrix and into the cytosol.^21^ We found that BDL-induced hyperammonaemia led to a more than 3-fold increase in cytosolic ROS generation rates (218.4 ± 67.6, n = 9, for control *vs*. 926.0 ± 67.7, n = 8, for BDL, *p* <0.0001; Fig. 4A, B, and C1 and C2), which was attenuated by OP treatment (474.9 ± 48.8, n = 11, *p* <0.0001; Fig. 4B and C3). Changes in the rate of ROS production from various exogenous or endogenous sources may play a role in redox signalling and have beneficial effects on cells.^22^ To investigate whether hyperammonaemia-induced ROS production in brain cells triggers pathological oxidative stress, we measured the rate of LP.

#### Hyperammonaemia increases the rate of lipid peroxidation and induces oxidative stress in the brain

**Fig. 5:**
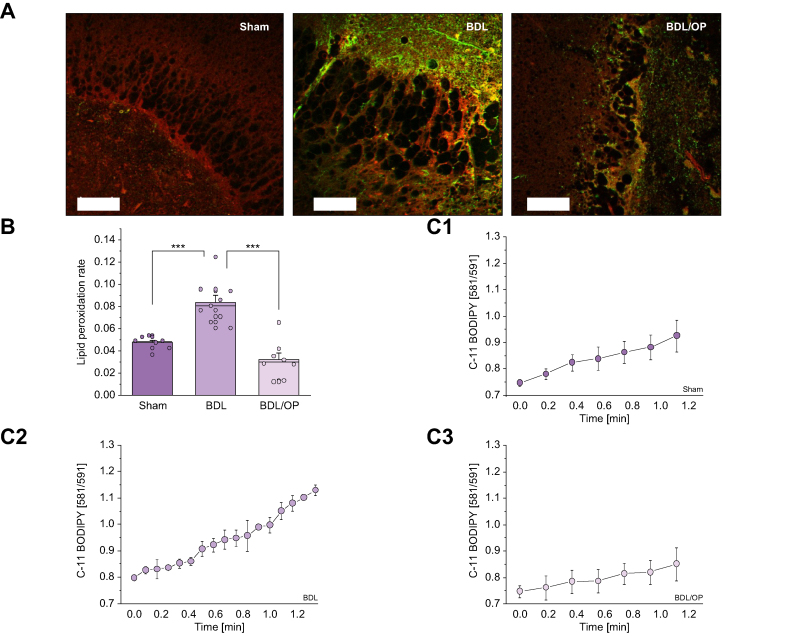
LP rate in acute slice of BDL rats is attenuated by OP. (A) Images of C11-BODIPY acute slices (red, non-oxidised; green, oxidised tissue) from sham-operated, BDL, and BDL/OP rats. (B) Quantification bar chart of LP rate in acute slices from sham-operated, BDL-subjected, and BDL/OP rats. (C) Mean representative traces of C11-BODIPY ratio from Sham operated (C1), BDL subjected (C2) and BDL/OP (C3) slices. Scale bar = 50 μm. Data are represented as mean ± SEM. ∗*p* <0.05, ∗∗*p* <0.001, ∗∗∗*p* <0.0001, two-tailed unpaired Student’s *t* test. BDL, bile duct ligation; LP, lipid peroxidation; OP, ornithine phenylacetate.

The rate of LP in acute brain slices was assessed using ratiometric fluorescent indicator C-11 BODIPY (Fig. 5A). In agreement with the results on ROS generation measurements, BDL-induced hyperammonaemia led to a significant increase in the rate of LP (0.047 ± 0.006, n = 7, for sham *vs*. 0.084 ± 0.019, n = 9, for BDL, *p* = 0.0003; Fig. 5B and C1 and C2). OP treatment efficiently protected the cells against LP (0.084 ± 0.006, n = 9, for BDL *vs*. 0.032 ± 0.006, n = 8, for BDL + OP, *p* <0.0001; Fig. 5B and C3). Thus, hyperammonaemia-induced overproduction of ROS in mitochondria and cytosol induced oxidative stress.

### *In vitro* and *ex vivo* evidence of neuronal cell death

**Fig. 6:**
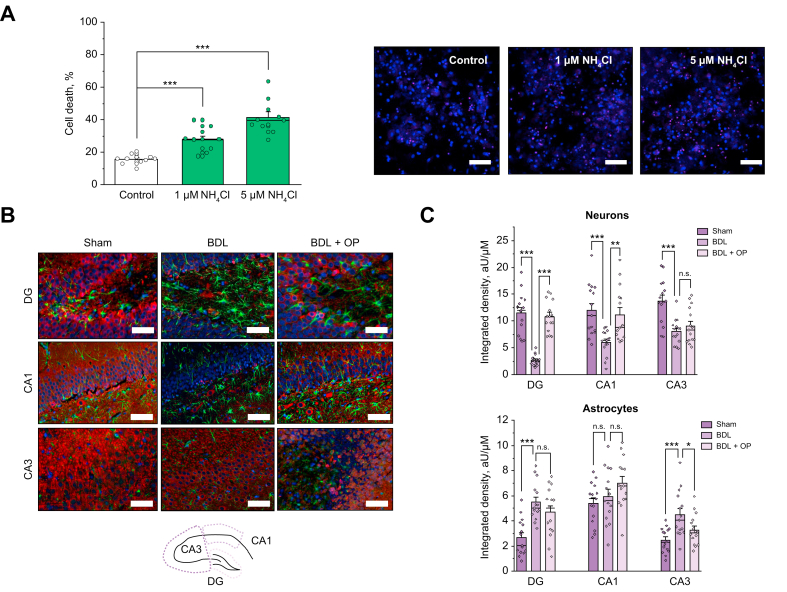
Cell death in *in vitro* and *ex vivo* models of hyperammonaemia. (A) Cell death rate assessed in primary co-culture of neurons and astrocytes upon the application of low (1 μM) and higher (5 μM) concentrations of NH_4_Cl (red: propidium iodide, non-viable cells; blue: Hoechst, total number of cells). (B) Immunostaining of neurons and astrocytes of fixed brain slices from sham-operated animals and those subjected to BDL and to BDL/OP from the DG, CA1, and CA3 areas from the rat hippocampus (green: GFAP, astrocytic marker; red: beta III tubulin, neuronal marker; blue: DAPI, cell nuclei). Scale bar = 250 μm. (C) Inset: schematic overview of the areas in rat hippocampus. Quantification bar charts for cell density in the DG, CA1, and CA3 areas for both neurons (upper chart) and astrocytes (lower chart). Data are represented as mean ± SEM. ∗*p* <0.05, ∗∗*p* <0.001, ∗∗∗*p* <0.0001, one-way ANOVA with Bonferroni correction. BDL, bile duct ligation; CA1, cornu ammonis 1; CA3, cornu ammonis 3; DG, dentate gyrus; GFAP, glial fibrillary acidic protein; OP, ornithine phenylacetate.

Oxidative stress induced by overproduction of mitochondrial ROS eventually results in mitochondrial dysfunction and the initiation of the process of cell death and neurodegeneration.^6^ To assess this, neuronal cultures (16DIV) were pretreated with low doses of NH_4_Cl (1 and 5 μM over 24 h). Cultures pretreated with 1 or 5 μM NH_4_Cl were found to indeed exhibit reduced cell viability (Fig. 6A and B, 27.8 ± 2.3%, n = 10, and 41.5 ± 3.7%, n = 10, respectively) compared with untreated cultures (15.7 ± 1.0%, n = 11, both *p* <0.0001).

*Ex vivo* cell density was assessed using immunofluorescence staining of neurons (labelled with beta III tubulin) and astrocytes (GFAP labelled) in the temporal lobe of the rat brains, that is, the hippocampal areas DG, CA1, and CA3. Significant neuronal loss was evident in all 3 areas: in the DG (2.68 ± 1.09%, n = 15 *vs*. 11.48 ± 0.99%, n = 15 for control, *p* <0.0001), in the CA1 (6.03 ± 0.61%, n = 15 *vs*. 12.03 ± 1.25%, n = 15 for control, *p* <0.0001), and in the CA3 (8.07 ± 0.60%, n = 15 *vs*. 13.77 ± 1.06%, n = 15 for control, *p* <0.0001) at 1 month after BDL (Fig. 6B and C, left and middle panel columns). This neuronal loss was significantly ameliorated by OP treatment in the DG (10.83 ± 0.76%, n = 15 *vs*. 2.68 ± 1.09%, n = 15 for BDL, *p* <0.0001) and CA1 (11.19 ± 1.28%, n = 15 *vs*. 6.03 ± 0.61%, n = 15 for BDL, *p* <0.0001) areas of the hippocampus (Fig. 6, right panel columns) but was not statistically significant in the CA3 area (n = 15, *p* = 0.3378).

Significant increase in the number of GFAP-positive astroglial cells was observed 1 month after BDL in the DG (5.51 ± 0.39%, n = 15 *vs*. 2.68 ± 0.37%, n = 15 for control, *p* <0.0001) and CA3 (4.51 ± 0.47%, n = 15 *vs*. 2.47 ± 0.26%, n = 15 for control, *p* <0.0001) areas, but not in the CA1 area (Fig. 6B and C, left and middle panels). It must be noted that reactive astrogliosis and glial scar formation was evident in the BDL brains. This effect on the astrocytes was only slightly affected by OP treatment (Fig. 6B and C, right panels).

## Discussion

This study was designed to evaluate the role of pathophysiologically relevant concentrations of ammonia on neuronal viability and the role of mitochondrial dysfunction and oxidative stress in neuronal toxicity in *ex vivo* brain slices from models of cirrhosis and in co-cultures of primary astrocytes and neurons. Our results strongly indicate that even low concentrations of ammonia (1–5 μM) can induce cell death in neurons (Fig. 6A). Importantly, also in an established animal model of low-grade hyperammonaemia,^23^ striking neuronal loss was observed in the hippocampal areas. Besides astrocyte swelling, profound neuronal injury and cell death were observed in cirrhotic rats. This might explain the recent observations suggesting that brain dysfunction following episodes of HE is not fully reversible.^12^^,^^13^ Our data underline the importance of early and targeted ammonia-lowering therapies in clinical practice and point to brain mitochondria as a potential novel therapeutic target in HE.

In both *in vitro* and *ex vivo* studies, we have shown that hyperammonaemia induces mitochondrial hyperpolarisation, increases ROS production, and induces LP. This is in agreement with previous studies that have shown that hyperammonaemia leads to increased generation of ROS in astrocytes,^8^^,^^24^^,^^25^ which is likely caused by ammonia-induced suppression of antioxidant enzymes such as catalase, glutathione peroxidase, and superoxide dismutase^26^^,^^27^ and interruption of astrocytic glutathione synthesis.^28^ Hyperammonaemia and oxidative stress may lead to an increase in cytoplasmic Ca^2+^, which affects mitochondrial function by inducing mPT, collapse of Δψm, osmotic swelling of mitochondrial matrix, uncoupling of oxidative phosphorylation and interruption of ATP synthesis.[29], [30], [31] It should be noted that neurons and glial cells have differences in mitochondrial metabolism and in redox balance. However, astrocytic dysfunction can lead to alteration in neuronal mitochondrial metabolism and in the production of major antioxidants that also can have implications for neuronal cell death.^32^

Most of the evidence for these previous observations was obtained in cellular models of hyperammonaemia. In this study, we have shown consistent results in a well-established, clinically relevant rodent model of minimal/nonovert HE.^33^ By applying live cell imaging techniques in the acute brain slices, the pathophysiological situation in these animals is very closely mimicked. Therefore, these data suggest that even when HE is not clinically evident, brain mitochondrial function is severely impaired. This is supported by our observations in mature cocultures of astrocytes and neurons, in which the application of a very low concentration of NH_4_Cl (5 μM) led to increased ROS production, LP, and a Δψm collapse. These are important observations, as novel treatments targeting mitochondria may be of benefit in minimal/non-overt HE and thereby potentially prevent the progression to overt HE.

The effect of the micromolar concentrations of ammonia on mitochondrial metabolism of neurons was dependent on the age of the primary cell cultures. Thus, the mechanisms of these effects were different – from inhibition of the mitochondrial respiration (NADH consumption) to mitochondrial uncoupling (Fig. 1). Differences in the effects between mature and immature neurons in culture can be possibly explained by the expression of the receptors in more mature neurons but more likely by the difference in the rate of ATP production and consumption in immature neurons.^15^ This finding provides a possible explanation for the clinical observation that older age is an independent risk factor for the development of HE.^34^

Another important observation in the present study was that of hyperammonaemia-induced neuronal loss. In the BDL model, we found significant neuronal loss in all 3 areas of the hippocampus, namely the DG, CA1, and CA3 regions of the brain, which was consistent with the observation of reduced cell viability in the neuronal cell cultures treated with NH_4_Cl. Most studies investigating hyperammonaemia-induced brain mitochondrial dysfunction have focussed on astrocytes, as these are traditionally thought to be the major cell type involved in brain ammonia metabolism. Our novel observation suggests that ammonia directly induces neuronal cell death, even at very low concentrations and in the rodent model of minimal HE. It was intriguing to note that although ammonia-lowering treatment with OP led to recovery of mitochondrial function in the acute brain slice studies, there was a persistence of neuronal injury and cell death supporting the previous observations that HE is not completely reversible.

This study has a few limitations that should be considered. Firstly, we were not able to measure plasma ammonia levels in the BDL rat model. This was because preparation of the acute brain slices does not allow the withdrawal of a clean, non-haemolysed blood sample. However, we have extensive experience with this BDL model, including OP treatment. Ammonia levels of large numbers of animals included in an identically performed BDL model have been previously published^18^ (Table S1) and showed small SDs. Secondly, the number of animals included in the current study was relatively low. Nevertheless, in keeping with the principles of the ARRIVE and 3Rs guidelines, it was considered unethical to proceed with inclusion of more animals, as highly statistically significant differences between groups were already observed. Finally, this study was not designed to investigate the underlying mechanisms of hyperammonaemia-induced ROS production, LP, and Δψm. Further studies are needed to explore this and to identify specific therapeutic targets to protect mitochondrial function during hyperammonaemia.

In conclusion, this study makes the novel observation that low-grade hyperammonaemia and minimal HE are associated with significant brain mitochondrial dysfunction, which results in increased ROS production, LP, and ultimately neuronal cell death. In addition, significant neuronal loss was observed in an animal model of cirrhosis with low-grade hyperammonaemia, which is only partially restored by correction of ammonia levels. These findings point towards the need for novel treatments targeting mitochondrial dysfunction, even in low-grade hyperammonaemia and minimal HE.

## Financial support

AJCK received funding from the 10.13039/501100009253EASL.

## Authors' contributions

Conceptualisation: PRA, AJK, AYA, RJ. Methodology: PRA, AJK. Validation: PRA, AJK. Formal analysis: PRA, AJK. Investigation: PRA, AJK. Data curation: PRA, AJK. Visualisation: PRA. Writing – original draft preparation: PRA, AJK. Writing – review and editing: PRA, AJK, AH, AnH, AYA, RJ. Technical and experimental support: AH, AnH. Resources: AYA, RJ. Supervision: AYA, RJ.

## Data availability statement

The data generated and analysed during the current study are included in this published article or available from the corresponding author upon reasonable request.

## Conflicts of interest

PRA, AJK, AH, AnH, and AYA have no conflicts to declare. RJ has research collaborations with Yaqrit and Takeda. He is the inventor of OPA, which has been patented by University College London and licensed to Mallinckrodt Pharma. He is also a founder of Yaqrit Limited, a spin out company from University College London. He has also cofounded Hepyx Ltd. and Cyberliver Ltd.

Please refer to the accompanying ICMJE disclosure forms for further details.
