# Supplementary material for: Hyperammonaemia induces mitochondrial dysfunction and neuronal cell death
Source: JHEP Rep. 2022 May 23;4(8):100510. doi: 10.1016/j.jhepr.2022.100510 (PMC9278080; doi:10.1016/j.jhepr.2022.100510)
Supplement: Multimedia component 1 [file mmc1.pdf]

Hyperammonaemia induces mitochondrial dysfunction and neuronal cell death

Plamena R. Angelova, Annarein J.C. Kerbert, Abeba Habtesion, Andrew Hall, Andrey Y. Abramov, Rajiv Jalan

Table of contents

Table S1..2

1.

Table S1. Plasma levels of ammonia, bilirubin and ALT in a BDL rat model with and without OP treatment as we previously reported in Hadjihambi et al. 2017.

**Parameters**	**Sham (n=22)**	**BDL (n=26)**	**BDL+OP (n=14)**
**Ammonia (umol/L)**	56±3	141±4	60±2
**Bilirubin (umol/L)**	5±0.5	205±3	161±6
**ALT (U/L)**	11±0.4	130±2	87±4
