# Supplementary material for: Hyperammonaemia induces mitochondrial dysfunction and neuronal cell death
Source: JHEP Rep. 2022 May 23;4(8):100510. doi: 10.1016/j.jhepr.2022.100510 (PMC9278080; doi:10.1016/j.jhepr.2022.100510)
Supplement: Multimedia component 2 [file mmc2.docx]

**Journal of Hepatology**

**CTAT methods**

Tables for a “Complete, Transparent, Accurate and Timely account” (CTAT) are now mandatory for all revised submissions. The aim is to enhance the reproducibility of methods.

- Only include the parts relevant to your study
- Refer to the CTAT in the main text as ‘Supplementary CTAT Table’
- Do not add subheadings
- Add as many rows as needed to include all information
- Only include one item per row

**If the CTAT form is not relevant to your study, please outline the reasons why:**

|  |
| --- |

- 1. **Antibodies**

| **Name** | **Citation** | **Supplier** | **Cat no.** | **Clone no.** |
| --- | --- | --- | --- | --- |
| Anti-beta III Tubulin antibody |  | Abcam | ab78078 | 2G10 |
| Anti-GFAP antibody |  | Abcam | ab7260 | n.a. |
| Goat Anti-Mouse IgG H&L (Alexa Fluor® 594) |  | Abcam | ab150120 | n.a. |
| Goat Anti-Rabbit IgG H&L (Alexa Fluor® 647) |  | Abcam | ab150079 | n.a. |

- 1. **Cell lines**

| **Name** | **Citation** | **Supplier** | **Cat no.** | **Passage no.** | **Authentication test method** |
| --- | --- | --- | --- | --- | --- |
| n.a. |  |  |  |  |  |

- 1. **Organisms**

| **Name** | **Citation** | **Supplier** | **Strain** | **Sex** | **Age** | **Overall n number** |
| --- | --- | --- | --- | --- | --- | --- |
| Rat |  | UCL breeding colony | Sprague‐Dawley | male and female | P0-2 | 4 |
| Rat |  | UCL breeding colony | Sprague-Dawley | male | 8-10 weeks | 10 |

- 1. **Sequence based reagents**

| **Name** | **Sequence** | **Supplier** |
| --- | --- | --- |
| n.a. |  |  |

- 1. **Biological samples**

| **Description** | **Source** | **Identifier** |
| --- | --- | --- |
| n.a. |  |  |

- 1. **Deposited data**

| **Name of repository** | **Identifier** | **Link** |
| --- | --- | --- |
| n.a. |  |  |

- 1. **Software**

| **Software name** | **Manufacturer** | **Version** |
| --- | --- | --- |
| ANDOR IQ | Andor, Belfast, UK | 3.0 |
| ZEN Blue | ZEISS, Germany | 3.4 |
| Image J | NIH, USA | 1.51j8 |
| OriginPro | OriginLab Copr., USA | 2019 |
| Prism | GraphPad Software, USA | 8 |
|  |  |  |
|  |  |  |

- 1. **Other (e.g. drugs, proteins, vectors etc.)**

| Ornithine Phenylacetate |  |  |
| --- | --- | --- |
|  |  |  |
|  |  |  |
|  |  |  |
|  |  |  |

- 1. **Please provide the details of the corresponding methods author for the manuscript:**

| 1. P.R.A: Brain slice preparation, primary culture preparation and live cell imaging 2. A.J.K.: Drug administration (OP), brain tissue collection and processing 3. An.H: Immunofluorescence staining of paraffin-embedded rat brains 4. A.H.: Bile duct ligation surgery |
| --- |

**2.0 Please confirm for randomised controlled trials all versions of the clinical protocol are included in the submission. These will be published online as supplementary information.**

| n.a. |
| --- |
